# Elevated preoperative platelet-to-lymphocyte ratio predicts poor prognosis of patients with primary gastrointestinal stromal tumor

**DOI:** 10.1186/s12876-020-01275-2

**Published:** 2020-04-22

**Authors:** Wei-Long Chang, Wen-Chang Yang, Xiang-Yu Zeng, Cheng-Guo Li, Zhen Xiong, Tao Wang, Rui-Zhi Zhang, Kai-Xiong Tao, Peng Zhang

**Affiliations:** 1 Department of Gastrointestinal Surgery, Union Hospital, Tongji Medical College, Huazhong University of Science and Technology, Wuhan, 430022 China; 2 Department of General Surgery, The First Affiliated Hospital of Zhengzhou University, Zhengzhou, 450052 China

**Keywords:** Gastrointestinal stromal tumor, Neutrophil-to-lymphocyte ratio, Platelet-to-lymphocyte ratio, Inflammatory biomarker, Prognosis

## Abstract

**Background:**

The neutrophil-to-lymphocyte ratio (NLR) and platelet-to-lymphocyte ratio (PLR) are considered to reflect the systemic inflammatory response and clinical prognosis. However, the independent prognostic values of the NLR and PLR for patients with gastrointestinal stromal tumor (GIST) remain debatable. This study aims to evaluate the prognostic value of preoperative NLR and PLR in GIST patients.

**Methods:**

We retrospectively reviewed all GIST patients diagnosed and surgically treated at Union Hospital between 2005 and 2018. The preoperative NLR and PLR were calculated to evaluate recurrence-free survival (RFS) and overall survival (OS) by Kaplan-Meier analysis. Univariate and multivariate Cox regression analyses were performed to estimate the independent prognostic values.

**Results:**

The median follow-up time was 49 months (interquartile range, 22–74 months). The preoperative PLR was significantly increased in the GIST patients with intermediate and high tumor risks. Increases in the NLR (≥2.34) and PLR (≥185.04) were associated with shorter RFS and OS (*P* < 0.01). Moreover, the multivariate analysis revealed that elevated PLR was an independent factor for shorter RFS (hazard ratio [HR]: 3.041; 95% confidence interval [CI]: 2.001–4.622; *P* < 0.001) and OS (HR: 1.899; 95% CI: 1.136–3.173; *P* = 0.014).

**Conclusions:**

The preoperative PLR is a potential biomarker of GIST and is related to the clinical outcome. An elevated preoperative PLR predicts poor prognosis of patients with primary GIST after complete surgical resection.

## Background

Gastrointestinal stromal tumor (GIST) is the most common mesenchymal neoplasm originating from the wall of the gastrointestinal (GI) tract; it accounts for 0.1 to 3% of all GI tumors and approximately 6% of all sarcomas [1]. GIST is most commonly located in the stomach (60%), followed in order by the small intestine (25%) and rectum (5%), and is also occasionally detected in the esophagus, mesentery, omentum and retroperitoneum [2]. Although GIST was once considered a leiomyoma or leiomyosarcoma, it is now distinguished from other mesenchymal tumors because it originates from interstitial cells of Cajal or their precursor cells and possesses a characteristic activating mutation in c-KIT or platelet-derived growth factor receptor α (PDGFRA) [3].

Although complete surgical resection remains a mainstay treatment for localized, primary GIST [4], the 5-year relapse rate is estimated to be 29.5% [5]. Currently, the tumor mitotic rate, size, location and tumor rupture are considered important independent factors predicting GIST recurrence [6], and postoperative adjuvant tyrosine kinase inhibitor (TKI) treatment may delay recurrence [7]. Therefore, an assessment of the risks of recurrence and progression of GIST has become increasingly important for patients, and studies exploring additional prognostic factors for recurrence risk stratification might increase the prognostic accuracy [8].

Over the last decade, cancer-related inflammation has been clearly established as playing a critical role in promoting tumor progression and metastasis [9]. In fact, inflammatory processes have been evaluated using widely available biomarkers, including macrophage-stimulating protein, C-reactive protein, or other hematological parameters, such as the neutrophil-to-lymphocyte ratio (NLR) or platelet-to-lymphocyte ratio (PLR) [10–12]. Among these inflammatory biomarkers, the preoperative blood NLR and PLR are negatively correlated with the prognosis of patients with solid tumors, including colorectal, pancreatic, non-small cell lung and ovarian tumors [13, 14]. Notwithstanding, investigations of the clinical prognostic value of NLR or PLR for GIST patients are limited and the results remain topic of intense debate [15, 16]. Regardless, studies including large samples or long-term investigations of the association of the NLR or PLR with outcomes in GIST patients are lacking, and the topic warrants further study.

In the present study, we examined the preoperative NLR and PLR to clarify whether these values are correlated with the clinical outcomes of patients with primary resectable GIST in a Chinese population.

## Methods

### Patient population

**Fig. 1:**
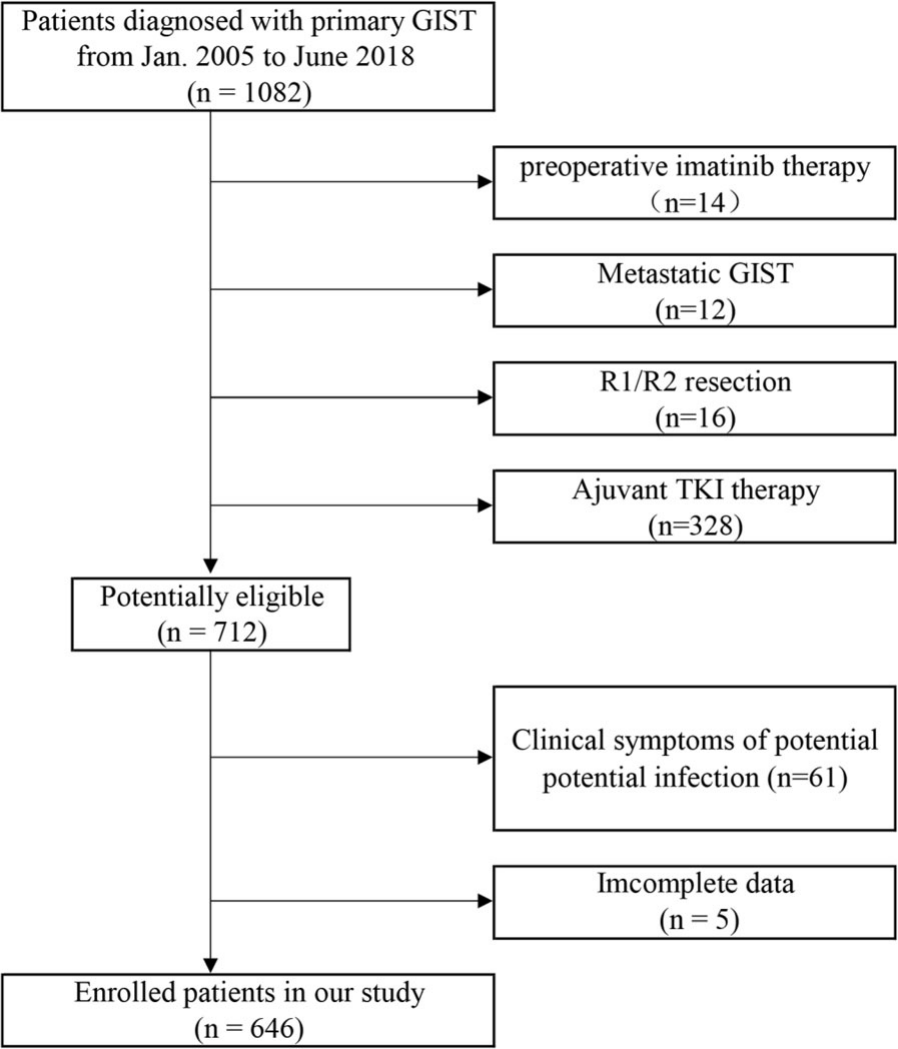
Flow chart showing the selection of objective patients

Between January 2005 and June 2018, 1082 primary GIST patients were diagnosed at Union Hospital, Tongji Medical College, Huazhong University of Science and Technology, Wuhan, China. According to the modified National Institutes of Health (NIH) consensus [17], 418 (38.6%) patients were at high risk, 115 (10.6%) at intermediate risk, 309 (28.6%) at low risk, and 240 (22.2%) patients at very low risk. The inclusion criteria were listed as follows: (1) without preoperative imatinib therapy, (2) with R0 resection, (3) without distant metastasis, (4) without adjuvant TKI therapy, (5) without clinical symptoms of potential infection, (6) with complete data. Finally, 646 patients were included in this study (Fig. 1). All enrolled patients were diagnosed with GIST relying heavily on CD117 and/or DOG1 immunohistochemical staining. Complete records for blood cell counts, population data and follow-up data were available for all patients. This retrospective study was approved by the Ethics Committee of Tongji Medical College, Huazhong University of Science and Technology. All methods were conducted in accordance with the approved guidelines.

### Peripheral blood counts

Preoperative peripheral blood samples were obtained from all patients within 7 days prior to surgery. No patient had clinical symptoms of an infection at the time of blood sampling. The blood NLR was defined as the absolute neutrophil count in the peripheral blood divided by the absolute lymphocyte count. Similarly, the blood PLR was defined as the absolute platelet count in the peripheral blood divided by the absolute lymphocyte count.

**Fig. 2:**
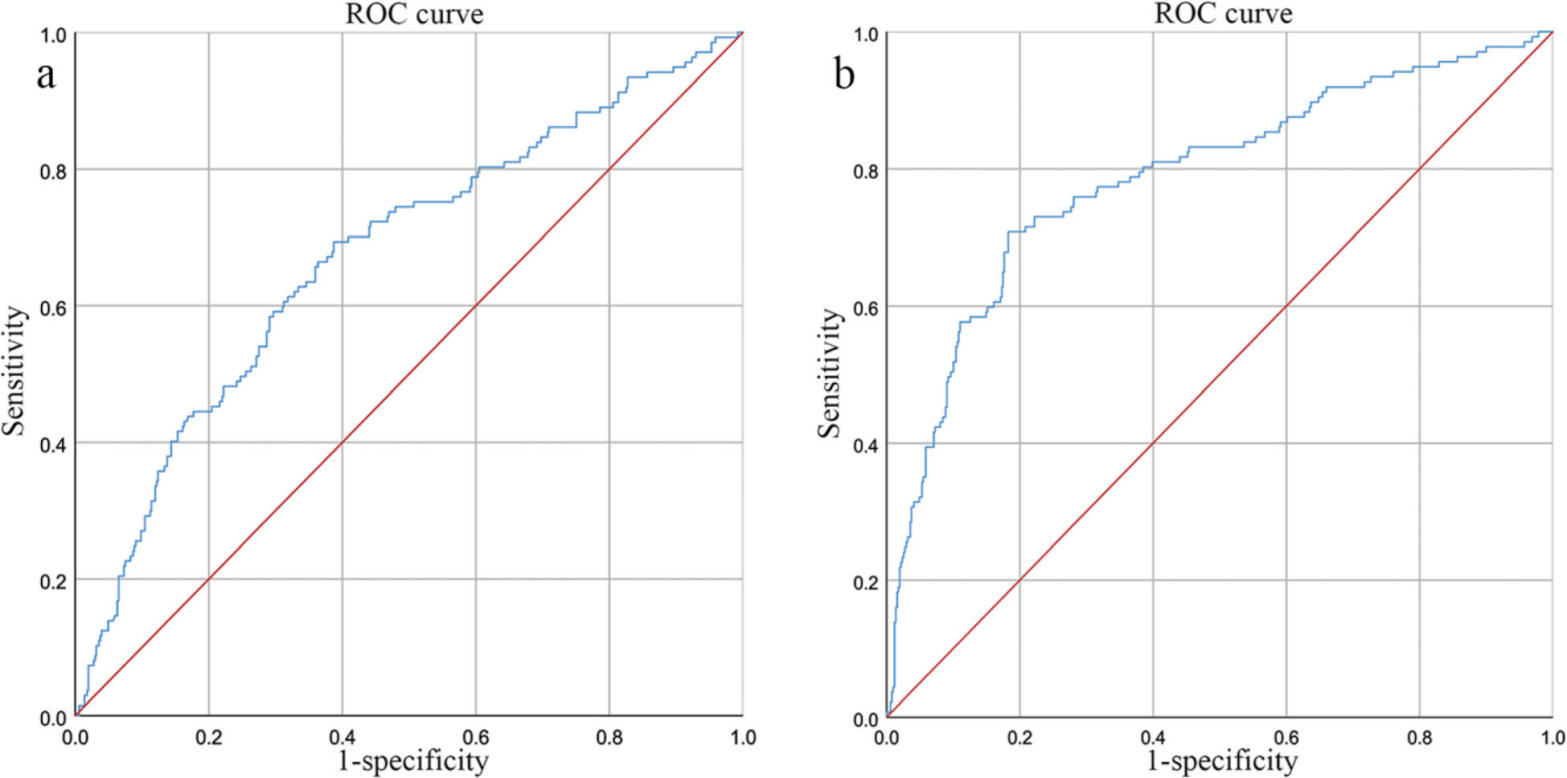
**a** Cut-off value for NLR applied with ROC curves for RFS. The area under the ROC curve was 0.673. An NLR value of 2.34 was considered as the cut-off value because of its maximal Youden index; the sensitivity and specificity were 69.3 and 70.8%, respectively **b**. Cut-off value for PLR applied with ROC curves for RFS. The area under the ROC curve was 0.788. An PLR value of 185.04 was considered as the cut-off value because of its maximal Youden index; the sensitivity and specificity were 70.8 and 81.7%, respectively

A receiver operating characteristic (ROC) curve analysis was performed to determine the cut-off values (with the highest specificity and sensitivity, also called the maximal Youden index) for the NLR and PLR based on the recurrence-free survival (RFS) data to define the high- and low-risk groups (Fig. 2). The high-risk group included patients with an NLR ≥2.34 and PLR ≥185.04.

### Data collection

The detailed clinical characteristics of all patients, including age, sex, tumor size, tumor site, mitotic index, preoperative absolute blood neutrophil count, absolute lymphocyte count, absolute platelet count and treatments received, were collected from the patient records and analyzed in our study.

Follow-up was conducted in the outpatient clinic or by telephone. The site, date and treatment of recurrence were also recorded, and the follow-up duration was calculated from the date of surgery to the date of the last follow-up or death. The data were finally censored at the last follow-up for the living patients.

### Statistical analysis

The summary statistics of the baseline characteristics of all enrolled patients who were, stratified by the NLR or PLR cut-off values, are reported as frequencies and proportions for categorical variables and as means ± SD for continuous variables. Categorical data were compared using the chi-square test. Continuous values were compared between two groups using two-tailed Student’s t-test or the Wilcoxon-Mann-Whitney U test. Pairwise correlations were evaluated using the two-tailed Pearson test. RFS was calculated as the time in months from tumor resection to pathological or radiological evidence of recurrence. Overall survival (OS) was defined as the time from the date of surgery to the date of death from any cause. Clinical outcomes were evaluated using Kaplan-Meier survival curves, and the groups were compared using the log-rank test. For the calculation of correlations, the NLR and PLR were first divided into dichotomous variables based on the cut-off value. Univariate and multivariate Cox proportional hazard regression models were used to evaluate associations with outcome variables. The level of statistical significance was set to *P* < 0.05 for all analyses. All tests were performed using SPSS statistical software (version 25, SPSS Inc., Chicago, IL, USA).

## Results

Six hundred and 46 patients, including 347 males and 299 females, were enrolled in this study cohort. All patients underwent complete surgical resection and refused the postoperative adjuvant imatinib therapy. The median age at diagnosis of the enrolled patients was 60 years (range, 23–91 years). The most common primary tumor location was the stomach (61.5%), followed by the jejunum and ileum (17.0%), duodenum (10.7%) and colorectum (5.0%). The median tumor size was 3.5 cm (range, 0.6–44 cm), and the median mitotic rate was 3 mitoses per 50 high-power fields (HPFs) (range, 0–51 mitoses). One hundred and 52 patients were at high risk, 71 at intermediate risk, 224 at low risk and 199 at very low risk. The median RFS and OS of the high PLR group were 83 months (95% confidence interval (CI): 68.6–97.4) and 124 months (95% CI: 102.3–145.8), respectively. In addition, the median RFS of the total patients and low PLR group were 144 months (95% CI: 126.1–161.9) and 156 months (95% CI: 133.7–178.3), respectively, and these values failed to reach the median OS.

Table 1 presents a comparison of the baseline demographic and clinicopathological features of the patients dichotomized by the NLR and PLR cut-off values. On the one hand, 354 patients (54.8%) had a low NLR, and 292 (45.2%) had a high NLR. A high NLR was significantly associated with the non-stomach location, tumor size and the tumor risk; however, it was not associated with age and the mitotic rate. On the other hand, 456 patients (70.6%) had a low PLR and 190 (29.4%) had a high PLR. Different to the findings for the NLR, a high PLR was associated with the non-stomach location, tumor size, the mitotic rate and the tumor risk.

**Table 1:** Clinical and pathological features of patients stratified by preoperative NLR and PLR

Characteristic	Low NLR	High NLR	*P* value	Low PLR	High PLR	*P* value
	(< 2.34)	(≥2.34)		(< 185.04)	(≥185.04)	
	(*n* = 354)	(*n* = 292)		(*n* = 456)	(*n* = 190)	
Age (years)	59.4 ± 11.9	59.2 ± 11.5	0.852	59.5 ± 11.0	58.7 ± 13.4	0.456
Gender						
Male	170	177	**0.020**	240	107	0.392
Female	184	115		216	83	
Primary tumor site						
Stomach	245	152	**< 0.001**	308	89	**< 0.001**
Duodenum	43	26		45	24	
Small intestine	32	78		56	54	
Colorectum	18	14		23	9	
Other	16	22		24	14	
Tumor size (cm)						
< 5	261	158	**< 0.001**	325	94	**< 0.001**
5–10	69	92		95	66	
> 10	24	42		36	30	
Unknown						
Mitotic rate (per 50 HPFs)						
≤5	313	242	0.110	408	147	**< 0.001**
6–10	31	35		38	28	
> 10	10	15		10	15	
Tumor risk						
Very low	139	60	**< 0.001**	159	40	**< 0.001**
Low	119	105		161	63	
Intermediate	37	34		55	16	
High	59	93		81	71	

Age is reported as mean years ± SD

*NLR* neutrophil-to-lymphocyte ratio, *PLR* platelet-to-lymphocyte ratio, *HPF* high-power field

Bold values indicate statistical significance at *P* < 0.05

**Fig. 3:**
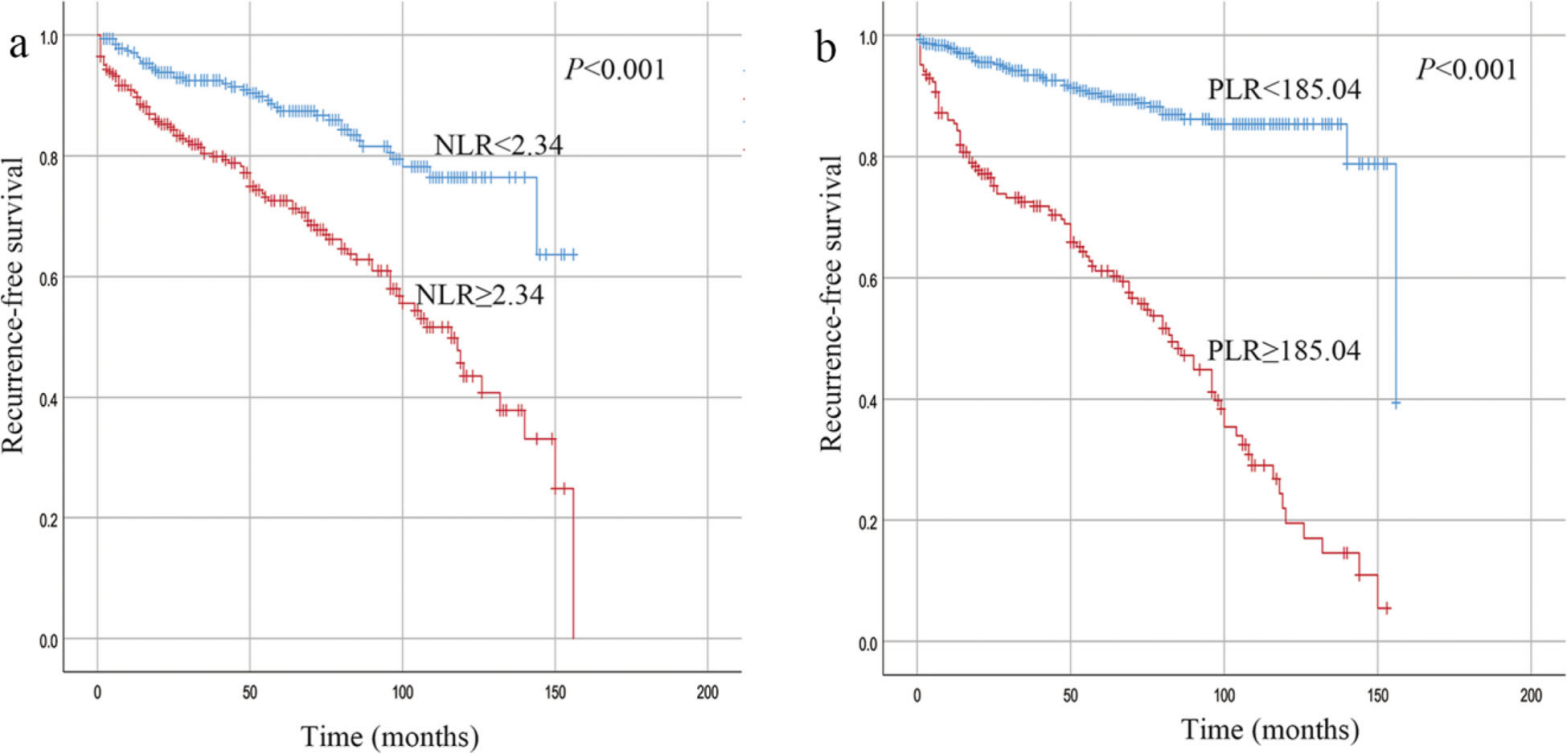
Kaplan-Meier curves for the RFS of GIST patients stratified according to a high or low NLR **a** and PLR **b**

**Fig. 4:**
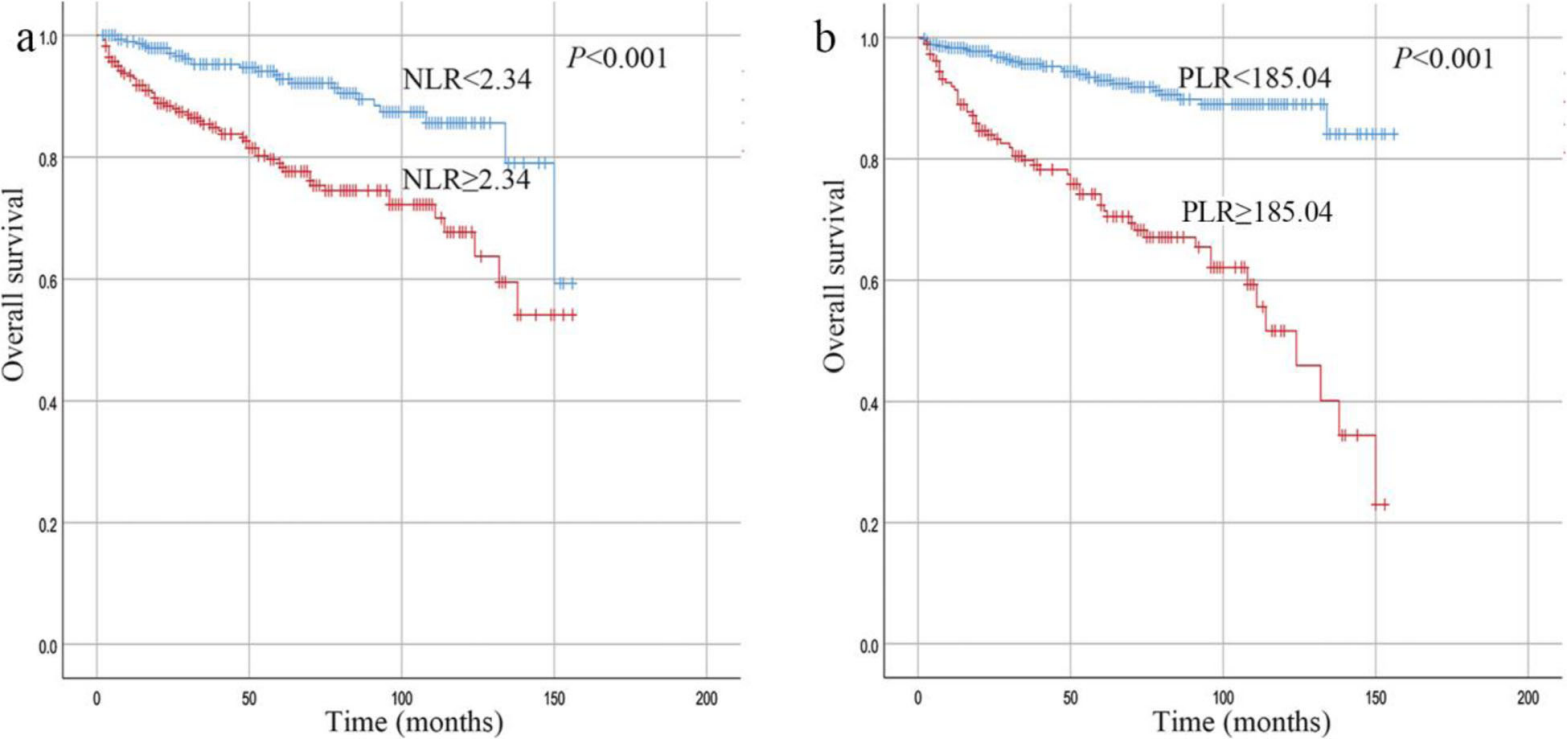
Kaplan-Meier curves for the OS of GIST patients stratified according to a high or low NLR **a** and PLR **b**

**Fig. 5:**
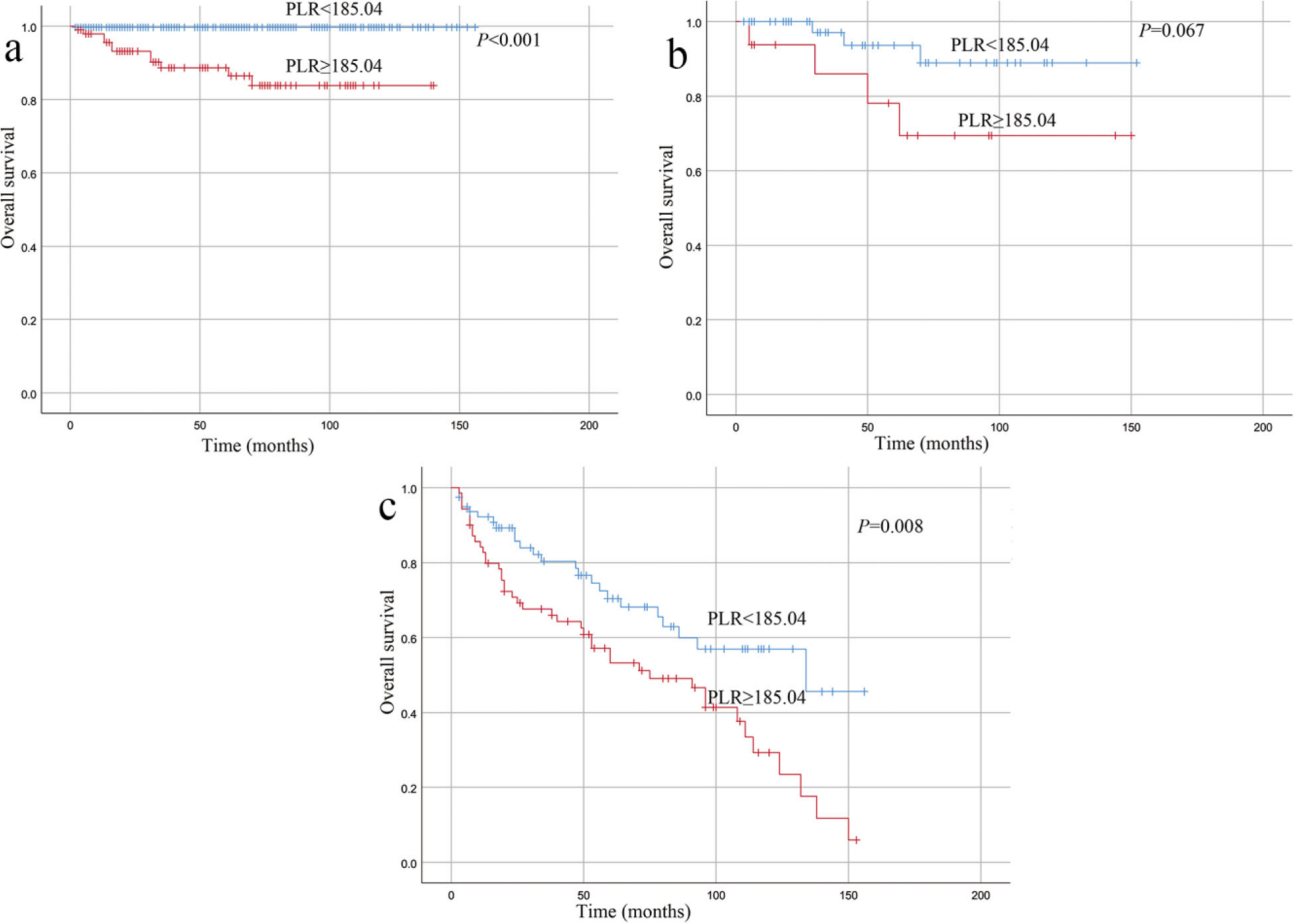
Kaplan-Meier curves for the OS of GIST patients in each risk group stratified according to a high or low PLR: **a** very low/low-risk GIST, **b** intermediate-risk GIST, **c** high-risk GIST

During a median follow-up period of 49 months (interquartile range, 22–74 months), 43 (12.1%) patients in the low NLR group and 95 (32.5%) in the high NLR group experienced recurrence. During the same period, 98 (51.6%) patients in the high PLR group and 40 (8.8%) in the low PLR group exhibited recurrence. Figure 3 shows the Kaplan-Meier curves for the RFS of the patients with a high or low NLR and PLR, and Fig. 4 shows the Kaplan-Meier curves for the OS of the patients with a high or low NLR and PLR. The follow-up data revealed that high preoperative NLR and PLR were both significant factors associated with a poor prognosis (*P* < 0.001). The elevated NLR and PLR suggested inferior RFS in each risk class group (*P* < 0.05, Table 2). Furthermore, the elevated PLR suggested inferior OS in the very low/low-risk group and high-risk group (*P* < 0.001 and *P* = 0.008, respectively; Fig. 5a and c). In addition, we observed a tendency that an increased level PLR resulted in an unfavorable OS in the intermediate-risk group (*P* = 0.067; Fig. 5b).

**Table 2:** Prediction of RFS and OS by NLR and PLR in different risk classes

Tumor risk	NLR		PLR	
	RFS	OS	RFS	OS
Very low and low	***P*** **= 0.003**	***P*** **= 0.012**	***P <*** **0.001**	***P <*** **0.001**
Intermediate	***P*** **= 0.015**	*P* = 0.068	***P <*** **0.001**	*P* = 0.067
High	***P*** **= 0.035**	*P* = 0.125	***P <*** **0.001**	***P*** **= 0.008**

*NLR* neutrophil-to-lymphocyte ratio, *PLR* platelet-to-lymphocyte ratio,

*RFS* recurrence-free survival, *OS* overall survival

Bold values indicate statistical significance at *P* < 0.05

Univariate and multivariate survival analyses of RFS and OS were performed to investigate whether the NLR or PLR was associated with clinical outcome. The univariate analysis revealed that a high NLR and high PLR indicated a poor prognosis for the RFS (Table 3) and OS (Table 4). Moreover, a multivariate analysis was performed using the Cox proportional hazards regression model to determine the independent prognostic significance of the NLR or PLR for determining the RFS and OS. The results showed that a high PLR was associated with a shorter RFS (hazard ratio [HR]: 3.041; 95% CI: 2.001–4.622; *P* < 0.001) and OS (HR: 1.899; 95% CI: 1.136–3.173; *P* = 0.014), as outlined in Tables 3 and 4. Furthermore, the mitotic rate, tumor size and PLR were determined to be independent prognostic factors for RFS and OS.

**Table 3:** Univariate and multivariate analyses to predict RFS

Variable	Univariate Analysis		Multivariate Analysis	
	HR (95% CI)	*P* value	HR (95% CI)	*P* value
Age (>60y)	1.025 (0.732, 1.434)	0.887		
Gender (male)	0.993 (0.709, 1.391)	0.969		
Location (non-stomach)	1.926 (1.373, 2.701)	**< 0.001**	0.965 (0.679, 1.372)	0.884
Tumor size (≥5 cm)	6.666 (4.399, 10.101)	**< 0.001**	3.228 (2.057, 5.067)	**< 0.001**
Mitotic rate (> 5)	8.317 (5.805, 11.406)	**< 0.001**	4.071 (2.825, 5.886)	**< 0.001**
NLR (≥2.34)	2.737 (1.903, 3.937)	**< 0.001**	1.302 (0.881, 1.925)	0.186
PLR (≥185.04)	6.331 (4.364, 9.184)	**< 0.001**	3.041 (2.001, 4.622)	**< 0.001**

*RFS* recurrence-free survival, *HR* hazard ratio, *CI* confidence interval,

*NLR* neutrophil-to-lymphocyte ratio, *PLR* platelet-to-lymphocyte ratio

Bold values indicate statistical significance at *P* < 0.05

**Table 4:** Univariate and multivariate analyses to predict OS

Variable	Univariate Analysis		Multivariate Analysis	
	HR (95% CI)	*P* value	HR (95% CI)	*P* value
Age (>60y)	1.116 (0.729, 1.708)	0.613		
Gender (male)	0.841 (0.548, 1.292)	0.430		
Location (non-stomach)	1.875 (1.222, 2.876)	**< 0.001**	0.951 (0.610, 1.484)	0.825
Tumor size (≥5 cm)	7.191 (4.172, 12.393)	**< 0.001**	3.079 (1.695, 5.593)	**< 0.001**
Mitotic rate (> 5)	13.163 (8.444, 20.520)	**< 0.001**	7.344 (4.536, 11.892)	**< 0.001**
NLR (≥2.34)	2.857 (1.792, 4.557)	**< 0.001**	1.507 (0.910, 2.496)	0.111
PLR (≥185.04)	4.907 (3.120, 7.718)	**< 0.001**	1.899 (1.136, 3.173)	**0.014**

RFS: recurrence-free survival, HR: hazard ratio, CI: confidence interval,

NLR: neutrophil-to-lymphocyte ratio, PLR: platelet-to-lymphocyte ratio

Bold values indicate statistical significance at *P* < 0.05

## Discussion

Since the Greek physician Claudius Galenus proposed the existence of similarities between tumor and inflammatory tissues approximately 1800 years ago [18], scientists have persistently explored their connections in various tumor types. For GIST patients with specific gene mutations, complete surgical resection and postoperative adjuvant TKI therapy have become the optimal treatment options. However, approximately 60% of patients relapse within 5 years after surgery [19]. Thus, other risk factors must also be considered to improve treatment decisions and patient outcomes. In particular, increasing evidence supports inflammation and the immune response as playing important roles in GIST [20, 21].

The NLR and PLR, biomarkers of the immune response, have been demonstrated to act as prognostic surrogates in various tumor types. However, no unanimous consensus exists for the potential predictive roles of NLR and PLR in GIST. Racz et al. [15] reported a predictive value for the PLR, but not NLR, while Jiang et al. [22] showed that the NLR was an independent prognostic factor. Thus, further investigation is required to probe this interesting question.

In the present study, we evaluated the efficacies of the NLR and PLR as preoperative inflammatory biomarkers in 646 Chinese patients who underwent surgery for primary GIST at our center and did not receive imatinib or sunitinib therapy over an observational period of 13 years. According to our analysis, the modified NIH criteria are valid and reliable for predicting the RFS and OS of Chinese GIST patients. Additionally, our findings provide evidence for the roles of the NLR and PLR as predictors of GIST recurrence. Although our study only revealed associations between the NLR and shorter RFS and OS in GIST patients, our finding is consistent with the results obtained in several other cancers, including hepatocellular carcinoma, cholangiocarcinoma, and colorectal cancer [23–25].

The prognostic role of the preoperative PLR in GIST patients has rarely been investigated until to date. Racz et al. [15] have confirmed the presence of a correlation between a high PLR and reduced RFS in North American patients. In the present study, we observed that a high preoperative PLR was an independent prognostic factor for reduced RFS and OS in primary localized GIST, consistent with the results obtained from patients with other malignant diseases, such as colon cancer and ovarian cancer. Thus, a PLR assessment may enable an accurate prediction of disease recurrence and patient survival after complete resection. Although the exact cause remains unclear, we propose that neutrophils are more sensitive to potential infection or inflammatory factors, which may lead to an NLR bias in GIST patients. In contrast, because the PLR is far more resistant to this effect, it may truly reflect the response to cancer-related inflammation. Furthermore, several inflammatory factors, including IL-6 and TNF-α, may stimulate megakaryocyte proliferation and induce reactive thrombocytosis [26]. Thus, the PLR increases in response to the imbalance in the immune response and impaired anti-tumor activity. In a previous study, the gastric infiltrating regulatory T cell count positively correlated with the tumor stage of gastric carcinoma, which is a marker of poor prognosis [27]. However, the interaction between peripheral blood cells and intratumor infiltration of immune cells requires further investigation in future studies.

Our study has some limitations. First of all, it was a retrospective study from a single center conducting the surgical management of Chinese GIST patients, although the conclusions have been drawn, prospective and appropriately designed studies are needed to verify the predictive value of these results. Secondarily, the cut-off values in our studies were defined based on a ROC curve analysis, and are different from other previous studies. Thus, a reasonable cut-off value that is useful for predicting prognosis of GIST patients should be identified for further investigation. In addition, the adjuvant TKI therapy cohort was excluded from this survival analysis because this treatment would significantly prolong the RFS and reduce the risk of recurrence [28]. Therefore, these patients may have a high preoperative NLR or PLR but a long RFS. Despite these limitations, to the best of our knowledge, our study represents a novel evaluation of the prognostic values of the preoperative NLR and PLR, which reflect the systemic inflammatory response, in Chinese patients with this relatively rare and complex disease who have undergone radical resection. A subsequent survival analysis revealed a relationship between these hematological ratios and the clinical prognosis. Unlike previous reports [15, 16], our findings indicated that an elevated PLR might serve as an independent predictor of GIST early recurrence and reduced survival; thus, a high PLR appears to be a reliable and strong prognostic predictor that may be used to guide therapy in the Chinese population. Furthermore, the modified NIH consensus classification performs well at predicting the survival of Chinese patients with GIST.

## Conclusions

In summary, a high PLR is a potential biomarker of GIST associated with a poor clinical prognosis. Due to its simplicity in predicting individual survival, a PLR of greater than 185.04 might independently refine the stratification of patients, indicating the need for more aggressive therapeutic approaches and more rigorous follow-up schedules. Future research conducted in multiple centers is needed to confirm the utility of PLR in predicting the prognosis of GIST patients.

## Data Availability

The datasets used and analyzed during the current study are available from the corresponding author upon reasonable request.

## Abbreviations

CI: Confidence interval
GI: Gastrointestinal
GIST: Gastrointestinal stromal tumor
HPFs: High-power fields
HR: Hazard ratio
NIH: National Institutes of Health
NLR: Neutrophil-to-lymphocyte ratio
OS: Overall survival
PLR: Platelet-to-lymphocyte ratio
RFS: Recurrence-free survival
ROC: receiver operating characteristic
TKI: Tyrosine kinase inhibitor
